# Internet- and mobile-based psychological interventions for sexual dysfunctions: a systematic review and meta-analysis

**DOI:** 10.1038/s41746-022-00670-1

**Published:** 2022-09-09

**Authors:** Anna-Carlotta Zarski, Julia Velten, Johannes Knauer, Matthias Berking, David Daniel Ebert

**Affiliations:** 1 Department of Clinical Psychology and Psychotherapy, Friedrich-Alexander-Universität Erlangen-Nürnberg, Erlangen, Germany; 2 Professorship Psychology and Digital Mental Health Care, Department of Sport and Health Sciences, Technical University Munich, Munich, Germany; 3 Mental Health Research and Treatment Center, Department of Clinical Psychology and Psychotherapy, Faculty of Psychology, Ruhr University Bochum, Bochum, Germany; 4 Department of Clinical Psychology and Psychotherapy, Institute of Psychology and Education, Ulm University, Ulm, Germany

**Keywords:** Therapeutics, Psychiatric disorders

## Abstract

Sexual dysfunctions are highly prevalent and undertreated. Internet- and mobile-based psychological interventions (IMIs) could be a promising addition to close this treatment gap, given their accessibility, anonymity, and scalability. This systematic review and meta-analysis investigated the efficacy of IMIs for sexual dysfunctions. A comprehensive literature search was conducted in August 2021 on randomized controlled trials investigating the effects of IMIs on sexual functioning and satisfaction compared to a control condition. Twelve RCTs with 14 comparisons were reviewed with six IMIs targeting female and six IMIs targeting male sexual dysfunctions and *n* = 952 participants were evaluated in the meta-analysis. IMIs were significantly more effective than control conditions (k = 11 waitlist control group, k = 3 online discussion board) at post-treatment for female sexual functioning (g = 0.59, CI: 0.28–0.90, I^2 ^= 0%) and satisfaction (g = 0.90, CI: 0.02–1.79, I^2 ^= 82%), and male sexual functioning (g = 0.18, CI: 0.02–0.34, I^2 ^= 0%). No significant effect was found for male sexual satisfaction (g = 0.69, CI: −0.13–1.51, I^2 ^= 88%) with substantial heterogeneity in studies. Most studies showed high dropout, with ten studies indicating some concern of risk of bias, and two studies showing high risk of bias. The results suggest that IMIs can be an effective treatment for sexual dysfunctions, although additional high-quality research is needed. Given the limited availability of specialized treatment for sexual dysfunctions and individual preferences for discrete treatment options, IMIs seem to be a valuable addition to routine care, empowering individuals to promote their sexual health on a guided self-help basis.

## Introduction

Sexual dysfunctions are a heterogeneous group of conditions that are characterized by a clinically significant impairment in a person’s ability to experience sexual pleasure or to respond sexually^1^. Sexual dysfunctions include female orgasmic disorder, female sexual interest/arousal disorder, genito-pelvic pain/penetration disorder, male hypoactive sexual desire disorder, premature ejaculation, delayed ejaculation, erectile disorder, substance/medication-induced sexual dysfunction, and other specific and unspecified sexual dysfunction^1^. Sexual dysfunctions are highly prevalent conditions^2,3^ associated with reduced quality of life^4^, impaired relationship satisfaction^5,6^, increased comorbidity of affective and anxiety disorders^7^, and substantial economic costs^8,9^.

Yet, less than half of women and men with a sexual condition seek help from a healthcare professional^10^. Despite progressive sexual education and reduced stigma, sexual conditions continue to be a taboo subject in many societies globally^11^. Fear of stigmatization, feelings of shame, and discomfort surrounding sexuality are major barriers to the utilization of available treatments^12–15^. Additional treatment barriers include low availability of specialized diagnostics and evidence-based treatments for sexual dysfunctions in routine care ﻿and extensive waiting lists for specialists^8^.

It is therefore unsurprising that individuals have been found to increasingly search the web anonymously for treatment options regarding disorders commonly perceived as sensitive and shameful, such as sexual dysfunctions^10,16^. As internet- and mobile-based interventions (IMIs) offer the advantage of scalable, anonymous, and easily accessible evidence-based treatment^17^, addressing sexual dysfunctions via the internet may be a particularly promising treatment avenue. IMIs also meet the preference of many individuals for self-help rather than on-site treatment^18^. Further, increased access to evidence-based treatments could help reduce the high number of reported unsuccessful treatment attempts for sexual dysfunctions^19,20^.

Although IMIs offer an exciting option to address reported barriers to treatment, research and implementation of IMIs for sexual dysfunctions is still in its infancy when compared to other mental disorders^21^. Despite research on an intelligent computer-based therapy system to diagnose and treat sexual dysfunctions in couples in the 1980s^22^, the field has not received an upsurge in further developments of IMIs as a result. The evidence to date on IMIs for sexual dysfunctions is summarized in one comprehensive meta-analysis for various mental disorders and two reviews. The meta-analytic findings from 2012 by Hedman and colleagues comprised four randomized controlled trials (RCTs) with five comparisons on solely female (k = 1), male (k = 3), or both sexual dysfunctions (k = 1)^23^. The within-group pre-post effect sizes varied widely between the studies (d = 0.21 to 1.52) with an average treatment effect of d = 0.67 (95%-CI: −0.25 to 1.59), showing moderate success. The IMI for female sexual dysfunctions achieved a large pre-post treatment effect of d = 1.52^24^, while IMIs for male sexual dysfunctions achieved small to medium effects between d = 0.21 and d = 0.52^25–27^. Based on these pre-post treatment results, IMIs were classified as a likely efficacious treatment option. Notably, to date, between-group effects have not been analyzed^23^. A meta-analysis from 2015 indicated that IMIs can improve sexual function and relationship satisfaction, but not distress levels in women. In men, there was a non-significant effect of IMIs on sexual function, but insufficient data to be able to draw any conclusions about the effects of IMIs on other outcomes^28^. A review exclusively on IMIs for female sexual dysfunctions from 2016^29^ showed an increase in research activity, with four completed RCTs^24,30–32^ of which one RCT included qualitative analyses only^31^ as well as one ongoing RCT^33^. Another review from 2018 focusing on IMIs for sexual health among individuals with cancer^34^ identified four relevant studies, of which three were RCTs^26,30,35,36^. No meta-analysis to date has analyzed the between-group effects of IMIs, so no comprehensive statement about its efficacy can be made to date.

Hence, there is a pressing need to synthesize the existing research on IMIs for female and male sexual dysfunctions to provide a comprehensive overview of the current research, to estimate the overall efficacy of this treatment approach in women and men, and to guide future research efforts. Thus, we conducted a systematic review and meta-analysis to examine whether women and men with sexual dysfunctions improved, on average, in their sexual functioning and sexual satisfaction when receiving an IMI compared to a control condition. These outcomes reflect the primary goals of psychological treatment for sexual dysfunction of establishing sexual functioning and increasing sexual satisfaction beyond reducing symptom severity^37^.

## Results

### Study selection

**Fig. 1:**
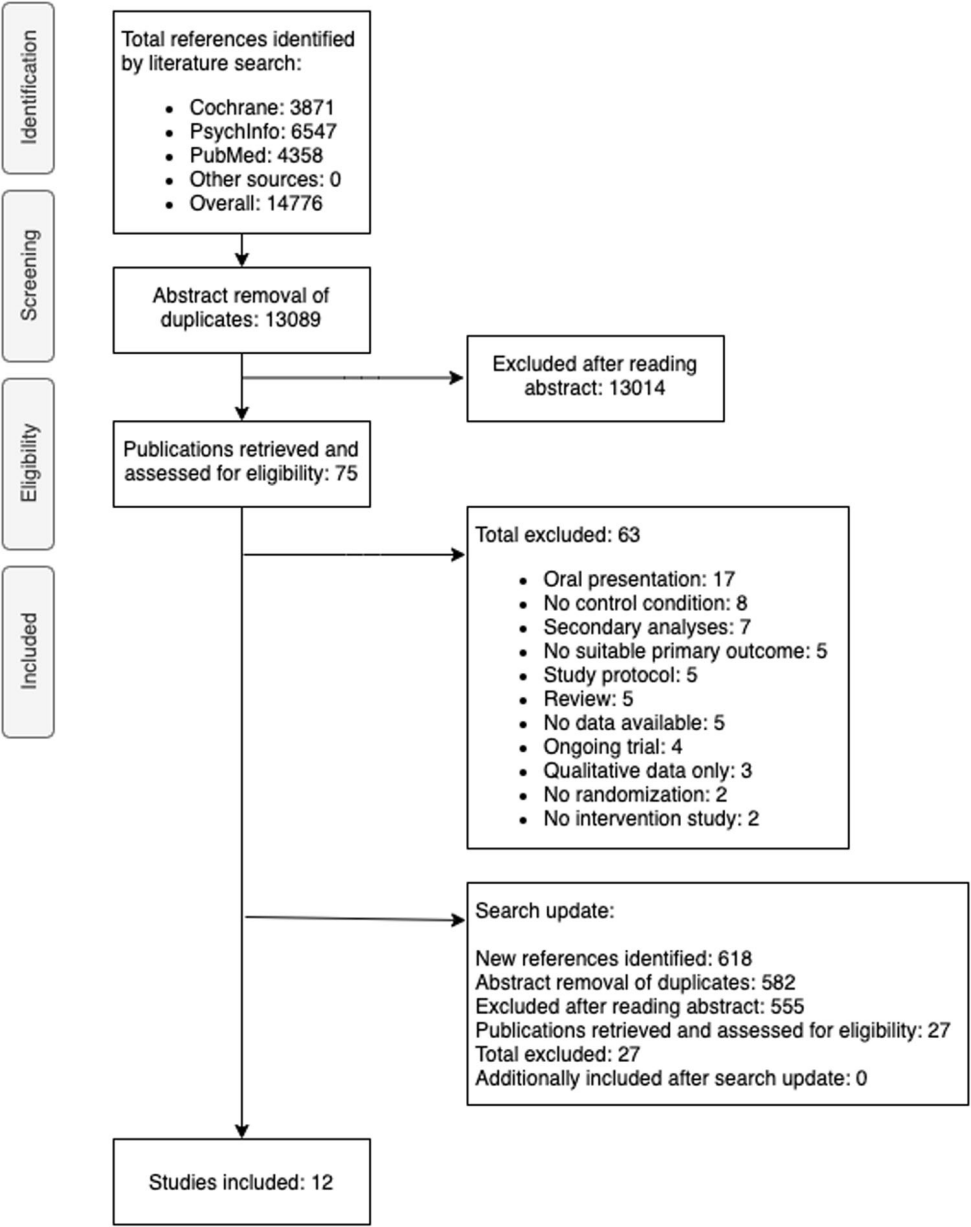
PRISMA flowchart.

A total of 13,089 articles were identified after removing duplicates. After abstract screening, 75 articles were identified for full-text review. Twelve articles reporting outcomes for 12 RCTs with 14 comparisons met the inclusion criteria of the study (see Fig. 1). A search update did not reveal any new eligible studies for inclusion in the study. The interrater reliability of the total selection between the two researchers was excellent (Cohen’s Kappa = 0.83)^38,39^. The study selection process and reasons for exclusion are displayed in Fig. 1.

### Characteristics of included studies

The characteristics of the 14 comparisons from 12 RCTs are described in Table 1 (references of included studies Supplementary References). The RCTs were conducted in Europe (k = 5, one from Sweden, two each from Germany and the Netherlands), Australia (k = 4), Canada (k = 1), Iran (k = 1), and the United States (k = 1). All studies were published between 2008 and 2021. One study received funding by a pharmaceutical company^27^. The 12 RCTs included a total of 1152 participants. Eleven studies included target outcomes for the meta-analysis evaluating *n* = 952 participants at post-treatment, 488 in the intervention and 464 in the control groups. Sample sizes at post-treatment ranged from *n* = 28 to *n* = 200 (median = 68). In six studies, the individuals affected by a sexual dysfunction were male, and in the other six studies, the affected individuals were female. The treatments were mainly focused on the affected person, but active participation of a sexual partner via couple exercises was mandatory in eight studies. In the other four studies, participation of a sexual partner was optional. Eight interventions were addressed at heterosexual patients only. All IMIs for male sexual dysfunctions targeted erectile dysfunctions, and in one case, additionally premature ejaculation. Three IMIs for female sexual dysfunctions were aimed at multiple, simultaneous sexual dysfunctions, including problems with desire, arousal, orgasm, and pain, whereas two IMIs specifically treated Genito-Pelvic Pain-Penetration Disorder, and one IMI targeted sexual distress associated with sexual difficulties. Of the 12 RCTs, five studies included samples in which sexual dysfunctions were related to a history of a medical disorder (prostate, gynecological, and breast cancer, and spinal cord injury). The average of mean age reported in 10 of the 12 RCTs was 41.55 years (SD = 10.77).

**Table 1:** Study descriptives.

Study	Gender	Target condition	Inclusion and exclusion criteria	Medical comor-bidities	Conditions	*n*; dropout post	Age (SD)	Primary outcome (measures), post-treatment measurement time	Secondary outcomes (measures)	Follow-up (duration)	Risk of bias	Country
Andersson et al., 2011	Male	Erectile dysfunction	IIEF-5: <21, partner (≥3 m, hetero-/homosexual); Excl.: medical cause, mental disorder, substance abuse	No	IMI (ICBT) Active condition (forum)	*n* = 39; 15% *n* = 39; 10%	57.62 (10.06), 55.50 (9.94)	Erectile function (IIEF-5), 7 weeks	Erectile function, orgasmic function, sexual desire, intercourse satisfaction, overall satisfaction (IIEF-15); relationship satisfaction (RAS); anxiety (BAI); depression (BDI-II); quality of life (WHOQOL-BREF);	Yes (IG, 6 m)	Some concerns	SW
McCabe et al., 2008	Male	Erectile dysfunction	Partner (heterosexual) Excl.: medical cause, mental disorder, substance abuse	No	IMI (Rekindle) waitlist control group	*n* = 24; 50% *n* = 20; 5%	NI	Erectile function, orgasmic function, sexual desire, overall satisfaction (IIEF-15), 10 weeks	marital satisfaction (KMSS); sexual satisfaction (ISS); self-esteem + relationship satisfaction (SEAR)	Yes (IG, 3 m)	Some concerns	AU
Schover et al., 2012	Male	Erectile dysfunction	Partner (heterosexual) (≥1 year)	Yes (prostate cancer)	IMI (CAREss) waitlist control group	*n* = 55; 51% *n* = 48; 10%	64 (7), NI	Erectile function (IIEF-15), female sexual functioning (FSFI), 12 weeks	distress (BSI-18); dyadic adjustment (A-DAS)	Yes (3, IG: 6, 12 m)	Some concerns	US
Van Lankveld et al., 2009	Male	Erectile dysfunction, premature ejaculation	Excl.: medical cause, mental disorder, substance abuse, severe relationship problems	No	IMI erectile dysfunction IMI premature ejaculation waitlist control group - erectile dysfunction waitlist control group - premature ejaculation	*n* = 30; 7% *n* = 22; 5% *n* = 28; 11% *n* = 18; 11%	Erectile dysfunction: 45.5 (13.0) Premature ejaculation: 40.1 (10.5)	Erectile function, sexual desire, overall satisfaction (IIEF-15); premature ejaculation (GRISS-PE), 12 weeks	sexual self-esteem + relationship satisfaction (SEAR-CONF), marital functioning (MMQ), impairment (GEQ)	Yes (IG, 3, 6 m)	Some concerns	NL
Wootten et al., 2017	Male	Erectile dysfunction	NI	Yes (prostate cancer)	IMI (My Road Ahead) IMI + forum Active condition (forum)	*n* = 47, 33 *n* = 48, 35 *n* = 47, 18 total: 27%	mean: 61 (7)	Erectile function, orgasmic function, sexual desire, intercourse satisfaction, overall satisfaction (IIEF-15), 10 weeks	depression + anxiety (DASS-21), quality of life (PC-QOL)	Yes (3, 6 m)	Some concerns	AU
Zarei et al., 2020	Male	Erectile dysfunction	Partner (heterosexual) Excl.: mental disorder, substance abuse, sex. dys. of partner	Yes (SCI)	App (SAMAR) waitlist control group	*n* = 35; 0% *n* = 35; 0%	36.7 (39.5) 35.3 (37.7)	Sexual adjustment (SAQ), sexual satisfaction (LSSS), marital adjustment (SMAQ), marital satisfaction (EMS), 8 weeks	NI	Yes (1, 2 m)	Some concerns	IRN
Classen et al., 2012	Female	Sexual distress	FSDS-R ≥ 24	Yes (﻿gynecologic cancer)	IMI (GyneGals) waitlist control group	*n* = 13; 23% *n* = 14; 0%	39.9 (29–58) 44.6 (28–59)	Sexual distress (FSDS-R), 16 weeks	Anxiety + depression (HADS), chronic disease (IIRS)	No	Some concerns	CAN
Hucker et al., 2015	Female	Multiple sexual dysfunction	Partner (heterosexual), Excl.: mental disorder, severe relationship problems	No	iCBT (Pursuing Pleasure) waitlist control group	*n* = 52; 58% *n* = 39; 46%	33.31 (7.4) 31.94 (5.17)	Female sexual functioning (FSFI), 11 weeks	Sexual distress (FSDR-R), erectile function (IIEF-15), premature ejaculation (PEDT)	Yes (IG, 3 m)	High	AU
Hummel et al., 2017	Female	Multiple sexual dysfunction	Sexual dysfunction DSM-IV diagnosis, Excl.: mental disorder, substance abuse, severe relationship problems	Yes (breast cancer)	IMI; waitlist control group	*n* = 84; 19% *n* = 85; 12%	51.6 (7.7) 50.5 (6.8)	Female sexual functioning (FSFI; SAQ), sexual distress (FSDS-R), relationship intimacy (PAIR), 24 weeks	Body image (QLQ-BR23), marital functioning (MMQ), menopausal symptoms (FACT-ES), anxiety + depression (HADS), HRQOL (SF-36)	Yes (IG, 3, 9 m)	Some concerns	NL
Jones et al., 2011	Female	Multiple sexual dysfunction	Partner (heterosexual)	No	IMI (Revive) waitlist control group	*n* = 26; 58% *n* = 27; 37%	34.91 (10.27) 33.30 (9.34)	Female sexual functioning (FSFI), 10 weeks	Sexual functioning (SFS), intimacy (PAIR), anxiety + depression (DASS-21)	Yes (IG, 3 m)	High	AU
Zarski et al., 2017	Female	Genito-pelvic pain-penetration disorder	Partner (>3 m, heterosexual) Excl.: medical cause, mental disorder, substance abuse	No	IMI, (Vaginismus-Free) waitlist control group	*n* = 40; 10% *n* = 37; 16%	25.83 (6.46) 28.95 (8.92)	Intercourse penetration ability (PEQ), 10 weeks	Non-intercourse penetration ability (PEQ), sexual fear (FSQ), female sexual functioning (FSFI), dyadic coping (DCI), treatment satisfaction (CSQ-I)	Yes (6 m)	Some concerns	G
Zarski et al., 2021	Female	Genito-pelvic pain-penetration disorder	Partner (heterosex.) Excl.: medical cause, mental disorder, substance abuse	No	IMI (Paivina-Care) waitlist control group	*n* = 100; 22% *n* = 100; 8%	29.46 (9.82) 28.04 (7.84)	Intercourse penetration ability (PEQ), 12 weeks	Non-intercourse penetration ability (PEQ), sexual fear (FSQ), female sexual functioning (FSFI), dyadic coping (DCI), relationship satisfaction (PFB-K), GAD (GAD-7), anxiety (STAI-T), quality of life (WHO-5), treatment satisfaction (CSQ-I), neg. ef. (INEP)	Yes (6 m)	Some concerns	G

Note: *A-DAS* dyadic adjustment scale – Abbreviated Form, *AU* Australia, *BAI* Beck anxiety inventory, *BDI-II* Beck depression inventory II, *BSI-18* brief symptom inventory-18, *CAN* Canada, *CGI-I* clinical global impression – Improvement Scale, *CSQ-I* client satisfaction questionnaire – internet-based interventions, *DASS-21* depression anxiety and stress scales – short version, *DCI* dyadic coping inventory, *EMS* ENRICH (evaluation and nurturing relationship issues, communication, and happiness) marital satisfaction scale, *Excl.* exclusion criteria, *FACT-ES* functional assessment of cancer treatment–endocrine symptoms, *FSDS-R* female sexual distress scale-revised, *FSFI* female sexual function index, *FSQ* fear of sexuality questionnaire, *G* Germany, *GAD-7* generalized anxiety disorder questionnaire, *GEQ* global endpoint question, *GRISS* Golombok rust inventory of sexual satisfaction, *GRISS-PE* Golombok rust inventory of sexual satisfaction - premature ejaculation subscale, *HADS* hospital anxiety and depression scale, *HRQOL* health-related quality of life, *IG* intervention group, *IIEF-15* international index of erectile functioning – 15 items version, *IIEF-5* international index of erectile functioning – 5 items version, *IIRS* illness intrusiveness ratings scale, *INEP* inventory for the assessment of negative effects of psychotherapy, *IRN* Iran, *ISS* index of sexual satisfaction, *KMSS* Kansas marital satisfaction scale, *LSSS* Larson’s sexual-satisfaction scale, *m* months, *MMQ* Maudsley marital questionnaire, multiple sexual dysfunctions = desire, arousal, orgasm, pain, *n* = sample size per group, *NI* no information, *NL* the Netherlands, *PAIR* personal assessment of intimacy in relationships inventory, *PC-QOL* prostate cancer-related quality of life scales, *PE* ﻿premature ejaculation, *PEDT* premature ejaculation diagnostic tool, *PEQ* primary endpoint questionnaire, *PFB-K* partnership questionnaire – short form, *QLQ-BR23* quality of life questionnaire – breast cancer module, *RAS* relationship assessment scale, *SAQ* sexual adjustment questionnaire, *SCI* ﻿spinal cord injury, *SD* standard deviation, *SEAR* self-esteem and relationship questionnaire, *SF-36* 36-item short form survey, *SFS* sexual function scale, *SMAQ* Spinner’s marital adjustment scale, *STAI-T* state-trait anxiety inventory – trait scale, SW Sweden, US United States, WHO-5 World Health Organization Well-Being Index, WHOQOL-BREF World Health Organization Quality of Life – Brief Version.

Eleven comparisons used an inactive control condition (waitlist control condition) while three comparisons used an active control condition (online discussion board). Five studies including male patients assessed sexual functioning with the International Index of Erectile Function (IIEF-15, IIEF-5^40,41^), and five studies assessed sexual satisfaction with the International Index of Erectile Function in four cases and the Larson’s sexual-satisfaction scale in one case^42,43^. Of those studies assessing sexual satisfaction, four considered it a primary outcome. Five studies including female patients assessed sexual functioning and sexual satisfaction using the Female Sexual Function Index (FSFI^44,45^), of which, three considered them as primary outcomes. Six studies included follow-up periods in both the intervention and the control condition, ranging from two to six months (median = 4.5 months); six studies conducted follow-ups in the intervention group only, ranging from 3 to 12 months (median = 6 months).

All psychological IMIs were based on the principle of Cognitive Behavioral Therapy (CBT) together with elements of sexual therapy including treatment components regarding: (1) psycho- and sexual education (k = 10), (2) cognitive restructuring (k = 8), (3) mindfulness (k = 1), (4) emotion regulation (k = 2), (5) relaxation (k = 4), (6) sensate focus (k = 9), (7) communication (k = 9), (8) relapse prevention (k = 6), (9) homework (k = 6) (10), and disorder-specific interventions (k = 4) such as exposure exercises, pain management for pain-penetration disorders, sexual exploration, and a decision-making algorithm to select suitable additional treatment for erectile dysfunction). IMIs for sexual dysfunctions associated with a medical disorder were also included (e.g., cancer-related topics). IMIs consisted of 3 to 10 modules (median = 5.5) that took between 8 and 24 weeks (median = 10.5) to be completed. Eleven interventions consisted of web-based modules delivered through a website or eHealth platform, and one intervention was smartphone-based. Three IMIs additionally included a moderated online discussion board (k = 2) or online chat groups (k = 1); one study included a smartphone-based diary. Ten studies involved some form of human guidance such as providing written feedback on completed modules (k = 5), moderation in an online discussion board or chat (k = 2), or on demand e-mail-based support (k = 3). The smartphone-based study did not report on any personalized guidance. Adherence reminders for module completion were included in nine studies, two of which also offered additional booster telephone calls. Ten studies provided at least one adherence measure, of which eight studies reported the percentage of participants who completed the IMI, which ranged between 8.11% and 87.00% (median: 53.26%).

### Risk of bias

Ten RCTs had an overall risk of bias with some concern, and two RCTs had a high risk of bias. Studies’ risk of bias was classified based on (1) the randomization process (k = 5 low RoB, k = 7 some concerns), (2) deviations from the intended interventions (k = 4 low RoB, k = 8 some concerns), (3) missing outcome data (k = 6 low RoB, k = 4 some concerns, k = 2 high RoB), (4) the measurement of the outcome (k = 12 some concerns), and (5) the selection of the reported results (k = 3 low RoB, k = 9 some concerns). RoB for each study is reported in Table 2 (risk of bias plot per study Supplementary Fig. 7).

**Table 2:** Intervention Content.

Study	Number of intervention modules	Duration of intervention	Adherence	Delivery modality	Guidance	Psycho- and sexual education	Cognitive restructuring	Mindfulness	Emotion regulation	Relaxation	Sensate Focus	Communication	Disorder-specific intervention	Relapse prevention	Homework
Andersson et al., 2011	7	7 w	3/37, 8.11%	Web-based modules	Written feedback on completed modules; email-based support on demand	Y	Y	N	N	Y	Y	Y	N	Y	Y
McCabe et al., 2008	5	10 w	12/24; 50.00%	Web-based modules	Email-based support on demand; adherence reminder	N	N	N	N	N	Y	Y	N	N	N
Schover et al., 2012	3	12 w	48/55, 87.27%	Web-based modules	Written feedback on completed modules; email-based support on demand; adherence reminder, 2 booster telephone calls	Y	Y	N	N	N	Y	Y	Y	Y	Y
Van Lankveld et al., 2009	NI	12 w	-	Web-based modules	Email-based support on demand	Y	Y	N	N	N	Y	N	N	N	N
Wootten et al., 2017	6	10 w	-	Web-based modules + online forum	Adherence reminder, automated feedback, moderation in online forum	Y	Y	N	Y	N	N	Y	N	Y	N
Zarei et al., 2020	NI	8 w	-	Mobile application	-	Y	NI	NI	NI	NI	NI	NI	NI	NI	NI
Classen et al., 2012	1	12 w	-	Web-based modules + online forum	90-min text-based chat session, moderation in online forum, adherence reminder	Y	N	N	Y	N	N	Y	N	N	N
Hucker et al., 2015	6	11 w	26/46, 56.52%	Web-based modules + online chat groups	Email-based support on demand; adherence reminder, moderation in online chat groups	Y	Y	Y	N	N	Y	Y	N	Y	Y
Hummel et al., 2017	4–5	max. 24 w	52/84, 61.90%	Web-based modules	Written feedback on completed modules; Email-based support on demand; adherence reminder, 2 telephone calls	Y	Y	N	N	Y	Y	Y	Y	Y	Y
Jones et al., 2011	5	10 w	17/26, 65.38%	Web-based modules	Email-based support on demand; adherence reminder	N	N	N	N	N	Y	Y	N	N	N
Zarski et al., 2017	10	10 w	13/40; 32.50%	Web-based modules	Written feedback on completed modules; email-based support on demand, adherence reminder	Y	Y	N	N	Y	Y	N	Y	N	Y
Zarski et al., 2021	8	12 w	43/100; 43.00%	Web-based modules + diary on smartphone	Written feedback on completed modules; email-based support on demand, adherence reminder	Y	Y	N	N	Y	Y	Y	Y	Y	Y

Note: Disorder-specific intervention = specific sexual therapy interventions such as vaginal training, w = weeks, adherence = defined as number and percentage of participants who completed treatment. Y yes, N no, NI no information.

### Main analyses

**Fig. 2:**
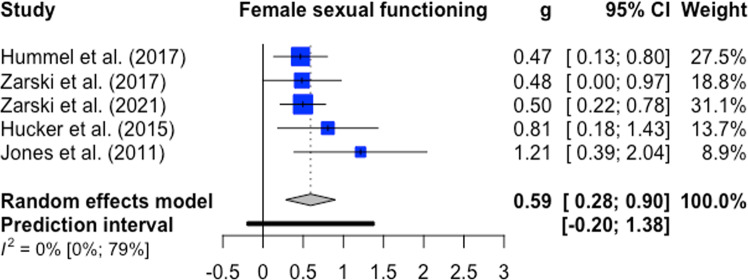
Forest plot for female sexual functioning outcomes. Note: All comparison groups were waiting control groups.

**Fig. 3:**
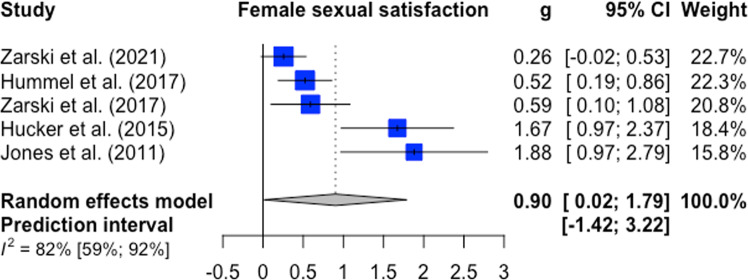
Forest plot for female sexual satisfaction outcomes. Note: All comparison groups were waiting control groups.

**Fig. 4:**
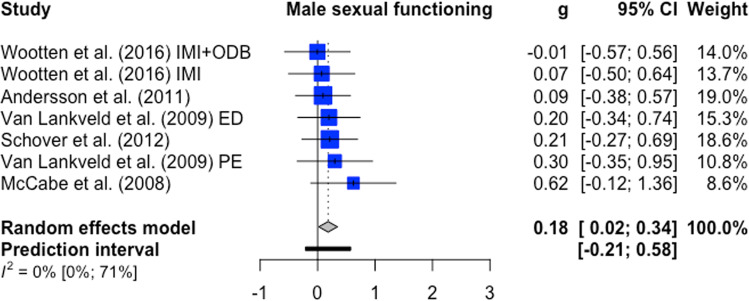
Forest plot for male sexual functioning outcomes. Note: ED erectile dysfunction, PE premature ejaculation; two comparisons included an active control condition (Andersson et al., 2011, Wootten et al., 2016).

**Fig. 5:**
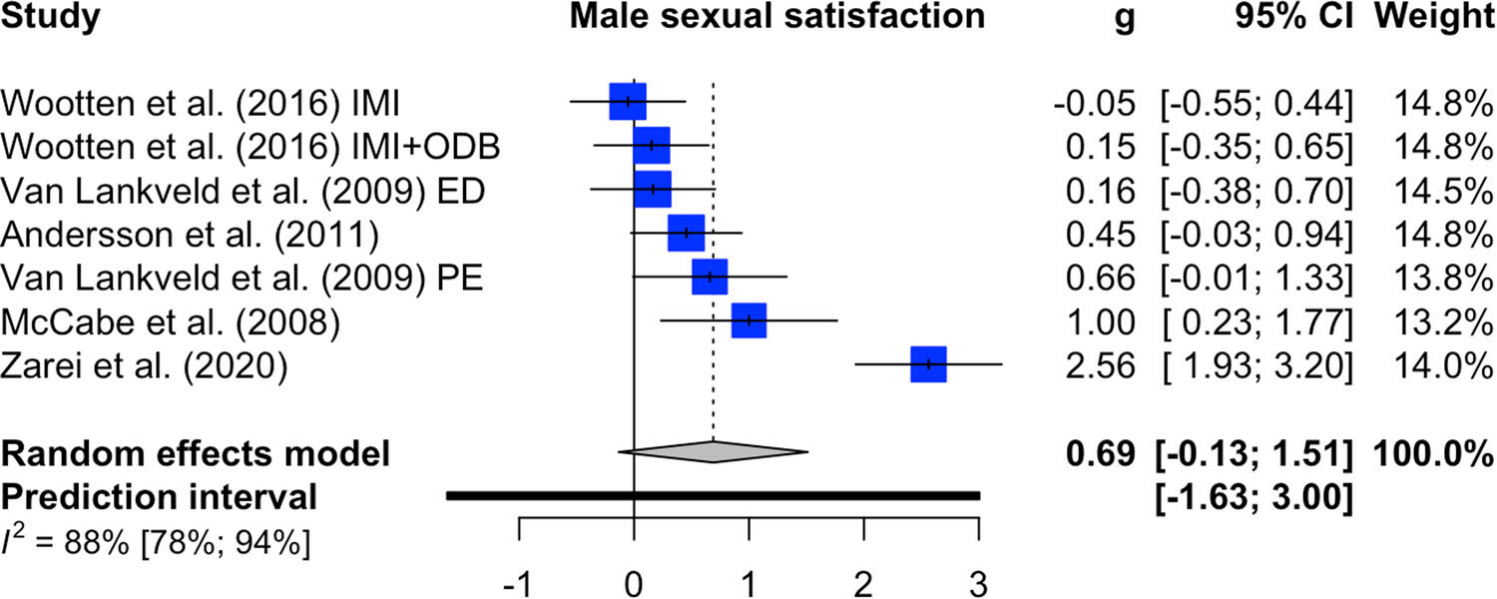
Forest plot for male sexual satisfaction outcomes. Note: ED erectile dysfunction, PE premature ejaculation, IMI IMI alone, IMI + ODF = IMI plus online discussion board; two comparisons included an active control condition (Andersson et al., 2011, Wootten et al., 2016).

Female sexual functioning and sexual satisfaction were both investigated in five comparisons (*n* = 482). The overall significant effect size for female sexual functioning was medium with g = 0.59 (95%-CI: 0.28–0.90, k = 5, *p* < 0.01, NNT = 3.09). Heterogeneity was low (I^2^ = 0%; 95%-CI: 0–79%) ﻿(forest plot Fig. 2). For female sexual satisfaction, the overall significant effect size was large with g = 0.90 (95%-CI: 0.02–1.79, k = 5, *p* = 0.0475; NNT = 2.10). Heterogeneity was considerable (I^2^ = 82%; 95%-CI: 59–92%) ﻿(forest plot Fig. 3). Male sexual functioning and male sexual satisfaction were investigated in seven comparisons (*n* = 363) and seven comparisons (*n* = 400), respectively. The overall significant effect size for male sexual functioning was small (g = 0.18, CI: 0.02–0.34, k = 4, *p* = 0.04; NNT = 7.46) with low heterogeneity (I^2^ = 0%; 95%-CI: 0–79%) (forest plot Fig. 4). The overall effect size for male sexual satisfaction was medium (g = 0.69; 95%-CI: −0.13–1.51, k = 5, *p* = 0.09; NNT = 2.67) and non-significant with considerable heterogeneity (I^2^ = 88%; 95%-CI: 78–94%) (forest plot Fig. 5).

### Sensitivity and subgroup analyses

Sensitivity analyses identified one outlier for male sexual satisfaction (Zarei et al., 2020). After omitting the outlier study, heterogeneity was reduced (I^2^ = 31%, 95%-CI: 0–72%), and the effect remained non-significant (g = 0.34; 95%-CI: −0.04–0.72, *p* = 0.07; NNT = 5.26). No statistical outlier was detected for the other outcomes. We did not conduct any subgroup analyses as the recommended number of studies per analysis was not reached.

### Publication bias

Egger’s test was significant for female sexual satisfaction (*p* = 0.01) and male sexual functioning (*p* = 0.04). Two studies were imputed in the trim-and-fill procedure for female sexual satisfaction resulting in a lower non-significant effect of g = 0.43 (95%-CI: −0.59–1.44, *p* = 0.35; NNT = 4.19). No study was imputed for male sexual functioning. Calculating *p*-curves was only possible for female sexual functioning and sexual satisfaction as two or less significant effects emerged in the main analysis for the other two outcomes in men. Significant right-skewed *p*-curves for female sexual functioning and female sexual satisfaction indicated that evidential value was present in both outcomes. The estimate of the true effect size in female sexual functioning resulted in a smaller effect size of d = 0.56 compared to the main outcome analysis. No adjusted estimate was calculated for the female sexual satisfaction outcome due to substantial heterogeneity (I^2^ = 87%) (Funnel plots and p-curves Supplementary Fig. 1–6).

## Discussion

Results indicated that IMIs are effective with medium to large effect sizes related to improved sexual functioning (g = 0.59) and satisfaction (g = 90) in women, and with small effect sizes related to improved sexual functioning in men (g = 0.18), compared to a control condition. Effects of IMIs on male sexual satisfaction were medium in size but not significant (g = 0.69). The findings were derived from 11 RCTs which included 952 participants with post-treatment data from seven countries. Heterogeneity of effect sizes varied from low for female and male sexual functioning to substantial for female and male sexual satisfaction. The median percentage of individuals who completed the IMI was 53%. The majority of studies showed some concern regarding risk of bias. There was evidence of publication bias, suggesting that unpublished studies with opposing effects may exist. All IMIs were based on the principles of CBT-based sexual therapy.

The medium to large effect sizes found in IMIs for female sexual functioning (d = 0.59) and satisfaction (d = 0.90) were comparable to the medium effect sizes found in face-to-face psychological interventions for female and male sexual dysfunctions on symptom severity (d = 0.58) and sexual satisfaction (d = 0.47) compared to waitlist conditions^37^. In contrast to face-to-face treatment, the effects of IMIs on male sexual functioning were smaller (g = 0.18) and non-significant for male sexual satisfaction (g = 0.69)^37^. One explanation for these divergent findings may be the fact that three comparisons in studies for men compared the IMI with an active control group in the form of an online discussion board. In an online discussion board, important common factors in sexual dysfunction such as mutual understanding and acceptance as well as destigmatization can take place through the exchange of patients among themselves even without specific therapeutic strategies being presented^46^. The overall lower effects and high heterogeneity in sexual satisfaction could be explained by differences among the IMIs in the nature and extent of the incorporation of dyadic aspects because sexual satisfaction has been previously found to be related to relational functioning^47^.

There are several limitations to be considered when interpreting the present findings. First, the number of identified RCTs was small. Despite an extensive systematic literature search, we cannot exclude the possibility that additional studies fulfilling the eligibility criteria exist. Moreover, publication bias could not be ruled out^48^. Most of the identified studies included small sample sizes and appeared to be pilot investigations, which limited the identification of small effects and restricted the generalizability of the findings. Thus, a number of the meta-analyses included only a small number of studies and were not adequately powered to detect clinically relevant differences between the IMI and the control conditions for all outcome measures. In addition, the small number of studies with small sample sizes meant that no further subgroup analyses could be performed^49^. Moreover, heterogeneity was substantial in most analyses but should be interpreted with caution due to the limited number of studies available^50^. Third, the majority of studies reviewed displayed at least an overall risk of bias with some concern (e.g., high study dropout rates), or no use of intention-to-treat analyses. Due to missing intention-to-treat data, calculations in most of the studies were performed with observed data, which might have led to an overestimation of the effect sizes. Thus, the results should be interpreted with caution. Fourth, most studies did not use diagnostic questionnaires with predefined symptom cut-offs or clinical interviews to establish a diagnosis of sexual dysfunction. Thus, variation in applied diagnostic criteria might limit the generalizability of the results to other populations with sexual dysfunctions. Fifth, we were not able to pool the long-term follow-up outcome data because only six studies included a long-term follow-up assessment in both the intervention and the control condition, and follow-up periods varied strongly among studies. Thus, no conclusions about the long-term efficacy could be drawn from the present study. It must, however, also be noted that the post-treatment measurement times also varied between the studies (range: 7 to 24 weeks).

More adequately powered studies are needed to investigate the efficacy of IMIs for sexual dysfunctions in women and men, people with sex variation (intersex), and gender-diverse communities, e.g., transgender and non-binary individuals, and individuals in same-gender and opposite-gender partnerships. Moreover, comprehensive diagnostics of sexual dysfunctions and associated conditions according to current guidelines should be included to determine the efficacy of IMIs for specific sexual dysfunctions and populations. Little is also known regarding which individuals are most likely to benefit from an IMI versus an in-person form of sexual therapy^51^. Investigating moderators of treatment efficacy for digital self-help and for other formats of sexual therapy can help to match affected individuals with the approach best suited to their needs. Most studies developed their program for heterosexual couples and examined their program in heterosexual samples. For this reason, the generalizability to sexual minorities and gender-diverse communities has been limited to date and should be the subject of further research. In this context, transcultural adaptation of IMIs for sexual dysfunction should also be explored^52,53^. Critically, a scientific investigation of potential negative outcomes and contraindications of treatment for sexual dysfunctions, irrespective of treatment modality, should also be conducted, which to date, has rarely been investigated. It is also unclear to what extent comorbid conditions such as anxiety or depression might complicate the treatment of sexual dysfunctions and which disorder should be addressed first^54^. Beyond clarifying who benefits from IMIs for sexual dysfunction, it has not yet been studied in detail which intervention components are responsible for positive treatment outcomes^55–58^. Due to their structured and standardized presentation of treatment components, IMIs are particularly well suited to investigate mechanisms of action and the significance of different components for treatment success^59^. Dismantling and additive-design studies, as well as studies comparing different treatment approaches, are needed to investigate different treatment components and approaches such as cognitive or mindfulness-based therapy^60^. The extent to which the efficacy of IMIs could be fostered by tailoring the number of treatment components and modules, individualizing support, and adapting the content more precisely to the respective situation or constellation of symptoms of the affected individuals, still remains an open research question^61^. We also need a better understanding of the specific mechanisms between guidance, adherence and outcomes as half of all individuals in the studies reviewed did not complete the IMI^62,63^. To increase the limited treatment effects, theory-based interventions are needed in order to confirm and further develop model assumptions^64^. While stepped treatment approaches starting with psychoeducation and sexual counselling are explicitly recommended, to the best of our knowledge, preventive approaches for sexual dysfunction have not yet been evaluated, and despite their accessibility, IMIs are often used after prolonged symptom duration. Moreover, many interventions have limited scope in terms of their target population and should be adapted and studied for different target populations regardless of sexual orientation or existing partnerships. Lastly, most of the existing IMIs are currently not available in routine care outside of research projects. Therefore, studies on the implementation of IMIs for sexual dysfunctions in routine care should be conducted to create tangible and accessible treatment options for the large number of affected individuals^64^.

Despite the stated limitations, especially concerning number and sample size of the RCTS identified, our findings indicated that sexual dysfunctions can be effectively treated with IMIs targeted towards female sexual functioning, female sexual satisfaction, and male sexual functioning. More high-quality research is needed to strengthen the evidence base for IMIs in the treatment of sexual dysfunctions and to generate knowledge to reach individuals who are most likely to benefit from an internet-based treatment. Nevertheless, the results indicate that IMIs for sexual dysfunction are ready to be implemented in routine care and more practice research is needed under routine conditions to accompany and advance implementation in health care systems.

## Methods

This systematic review was conducted in accordance with the Preferred Reporting Items for Systematic Reviews and Meta-Analyses (PRISMA) guidelines and the Cochrane Handbook for Systematic Reviews of Interventions^65–67^. The study was registered in the Open Science Framework (OSF; https://osf.io/m8kng).

### Eligibility criteria

Our eligibility criteria included a broad assessment of psychological IMIs for sexual dysfunctions among diverse populations. We included (1) peer-reviewed (2) randomized controlled trials using (3) a computer, internet- or mobile-based (4) psychological intervention as the primary treatment, (5) aiming to reduce symptoms of sexual dysfunctions in adult female or male individuals or couples (6) compared to an active or inactive control condition (7) at post treatment, with (8) symptom severity, sexual functioning, or sexual satisfaction as outcomes. We did not exclude studies based on participants’ age, sex, or any other demographic characteristic; comorbid general medical disorders were also not used as an exclusion criterion. Studies evaluating blended interventions (i.e., combined IMIs and face-to-face treatment), telemedicine, treatments in which the internet was only used for communication between therapist and patient, or those equivalent to face-to-face therapy (e.g., video therapy) were excluded. RCT arms including other treatments such as face-to-face psychotherapy, pharmacological, or physical therapies were excluded. No language restrictions were applied apart from formulating the search string in English.

### Search strategy

A systematic literature search was completed (until September 5th, 2020 with a search update covering the period up to August 3rd, 2021) in three major bibliographical databases (﻿Cochrane Central Register of Controlled Trials, PsycInfo, PubMed). Key search terms included a combination of terms indicative of sexual dysfunctions and internet- and mobile-based psychological treatment (Search String Supplementary Methods). Additionally, references in identified studies were checked for earlier publications, and authors were contacted in case further information was necessary to clarify study eligibility. The interrater reliability was assessed with Cohen’s Kappa for the initial agreement on full text eligibility with values <0 indicating no agreement, 0.00–0.20 none to slight, 0.21–0.40 fair, 0.41–0.60 moderate, 0.61–0.80 substantial, and 0.81–1.00 indicating excellent agreement^38,39^.

### Study selection

After removing duplicates, titles and abstracts of identified studies were first screened for potential eligibility. Second, full texts of potentially eligible studies were reviewed for inclusion criteria. Study selection, subsequent full text screening, rating for eligibility, and data extraction were performed independently by two researchers (J.K. & A.-C.Z.). All discrepancies were resolved by consensus with an additional third and fourth researcher (D.D.E. & J.V.).

### Data extraction

Extracted data included (1) bibliographical data (first author, year), (2) study design features (target condition, inclusion and exclusion criteria, study conditions, intervention name, type of control group (inactive control condition such as waitlist control conditions and active control condition such as attention control conditions), sample size, primary and secondary outcomes, outcome measures, follow-up, duration of follow-up, country of origin), (3) intervention characteristics (number of treatment modules, duration, treatment adherence, delivery modality, guidance, type of guidance, treatment components), (4) sample characteristics (gender, age, medical comorbidity), and (5) data for calculation of effect sizes (means and dispersion measures, preferably intention-to-treat post-treatment data, study dropout rates, handling of missing data). If data was not retrievable from the publication, study authors were contacted for clarification.

### Quality assessment

We evaluated the validity of included studies using the five domains of the risk of bias 2.0 tool by the Cochrane Collaboration, based on five criteria: (1) the randomization process, (2) deviations from the intended interventions, (3) missing outcome data, (4) outcome measurement, and (5) selection of the reported results (Sterne et al., 2019). Following the guidelines and related decision trees, the overall risk of bias was judged with either “low”, “some concerns”, or “high”. Given the difficulty of blinding participants and study staff in psychological intervention RCTs with non-interventional control conditions, the judgement guidelines for domain specific risk of bias were adjusted. Risk of bias assessment was conducted independently by two researchers (J.K. & A.-C.Z.).

### Statistical analyses

#### Meta-analytic procedure

The meta-analysis was performed using the *meta*^68^, *metafor*^69^, and *dmetar*^70^ packages in R version 3.6.2 (R Core Team, 2017). A random-effects pooling model was applied as considerable heterogeneity among studies was expected. For each comparison between an IMI and a control condition at post-treatment, we calculated the between-group effect size Hedges’ g (g) to adjust for small sample bias^71^, the 95% confidence interval (95%-CI), and the *p*-value (*p*) for each target outcome. In line with recommendations from Cohen (1988), Hedges’ g was interpreted with an effect size of 0.2 being considered small, 0.5 moderate, and 0.8 being considered a large effect^72^. Prediction intervals (95%) were calculated around the pooled effect sizes, indicating the interval within which the effect size of a similar future study would fall if selected at random from the same population of studies presently investigated^73^. Additionally, the effects were transformed into numbers-needed-to-treat (NNT) using the formula by Kraemer and Kupfer to indicate the number of patients that need to be treated to gain one additional positive outcome in comparison to a control condition^74^. Heterogeneity was evaluated using the I² statistic and its 95% confidence interval (Higgins & Thompson, 2002). I² heterogeneity of 25% can be regarded as low, 50% as moderate, and 75% as substantial heterogeneity^75^.

#### Main analyses

In the main outcome analyses, we pooled studies with the same target outcome of female or male sexual functioning or sexual satisfaction to generate a mean effect size for each investigated outcome. We pooled all target outcome data of eligible studies for the respective analysis, irrespective of whether the measure was defined as a primary or secondary outcome. If trials were multi-armed, reporting two or more intervention groups to the same control comparison, we divided the sample size of the comparison to avoid inflating power^76^.

#### Sensitivity and subgroup analyses

To assess the influence of individual studies and potential outliers on the overall effect, we conducted “leave-one-out” analyses. We first identified significant outlying studies exerting the greatest influence on the results. In case of significant outliers, we recalculated the analyses with the outlier removed^77^. Subgroup analyses to investigate potential sources of heterogeneity were only performed if the meta-analysis contained at least k = 10 studies per research question following the recommendation by Schwarzer and colleagues^78^.

### Publication bias

Indications of publication bias were evaluated via two approaches based on different assumptions of the origin of the bias. Assuming that publication bias operates through effect sizes, we investigated the funnel plot visually^79^ and tested for asymmetry via Egger’s test^80^. If the test for asymmetry was significant, indicating publication bias, Duval and Tweedie trim-and-fill procedure was used to adjust for possible bias by obtaining an estimation of the pooled effect when accounting for missing studies^81^. Alternatively, *p*-curve analyses were applied as a second approach, assuming that the publication bias was mainly driven by statistical significance^82,83^. A significant test of right‐skewness of the *p*-curve indicates the presence of evidential value in an analysis. To calculate *p*-curves, three or more significant effects were needed^70^. We only reported the adjusted effect size estimate for analyses with *I*^*2*^ < 50%, as a lack of robustness has been noted for analyses with substantial heterogeneity^84^.

### Reporting summary

Further information on research design is available in the Nature Research Reporting Summary linked to this article.

## Supplementary information

Supplementary Information

Reporting Summary

## Data Availability

Data collected and used in this meta-analysis can be requested from the corresponding author.
