# Supplementary material for: Internet- and mobile-based psychological interventions for sexual dysfunctions: a systematic review and meta-analysis
Source: NPJ Digit Med. 2022 Sep 9;5:139. doi: 10.1038/s41746-022-00670-1 (PMC9463146; doi:10.1038/s41746-022-00670-1)
Supplement: Supplementary file 1 — Supplementary Information [file 41746_2022_670_MOESM1_ESM.pdf]

Supplement:

Internet- and Mobile-Based Psychological Interventions for Sexual Dysfunctions: A Systematic Review and Meta-Analysis

Anna-Carlotta Zarski[1,2]*, Julia Velten[3], Johannes Knauer[4], Matthias Berking[1], David Daniel Ebert[2]

[1] Department of Clinical Psychology and Psychotherapy, Friedrich-Alexander-Universität Erlangen-Nürnberg, Erlangen, Germany

[2] Chair for Psychology and Digital Mental Health Care, Department of Sport and Health Sciences, Technical University Munich, Germany

[3] Mental Health Research and Treatment Center, Department of Clinical Psychology and Psychotherapy, Faculty of Psychology, Ruhr University Bochum, Germany

[4] Department of Clinical Psychology and Psychotherapy, Institute of Psychology and Education, Ulm University, Germany

*Corresponding Author

Anna-Carlotta Zarski

Department of Clinical Psychology and Psychotherapy

Friedrich-Alexander-Universität Erlangen-Nürnberg

Nägelsbachstr. 25a

91052 Erlangen, Germany

E-mail: anna-carlotta.zarski@fau.de

Supplementary Figures

Supplementary Figure 1. Funnel plot female sexual functioning outcome

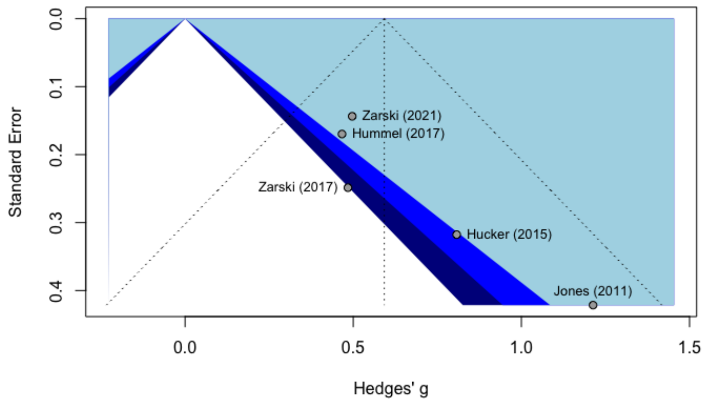

Supplementary Figure 2. Funnel plot female sexual satisfaction outcome

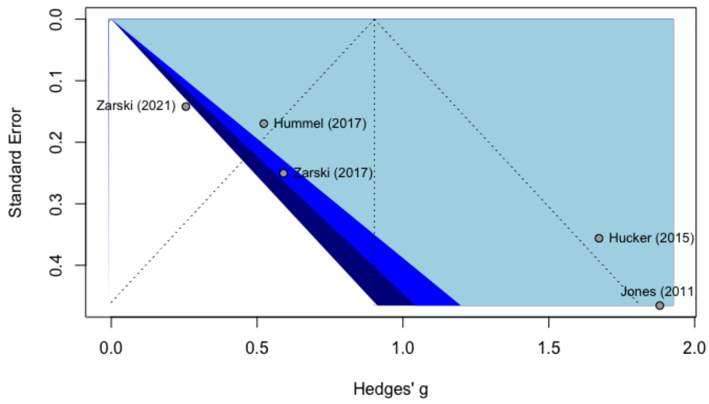

Supplementary Figure 3. Funnel plot male sexual functioning outcome

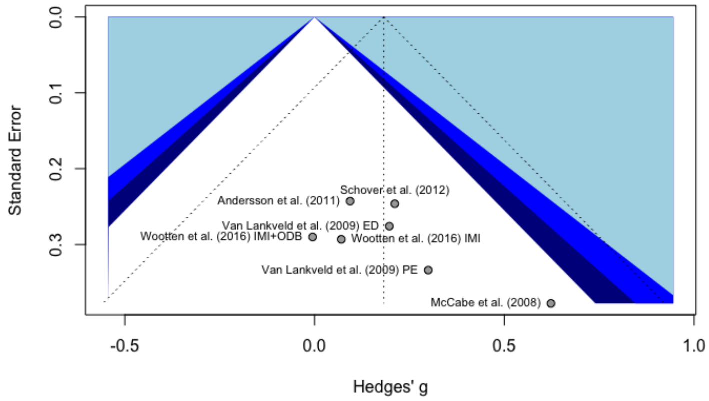

Supplementary Figure 4. Funnel plot male sexual satisfaction outcome

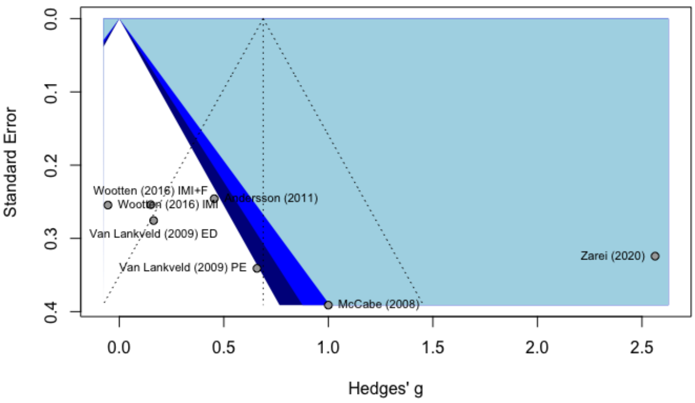

Supplementary Figure 5. P-curve female sexual functioning

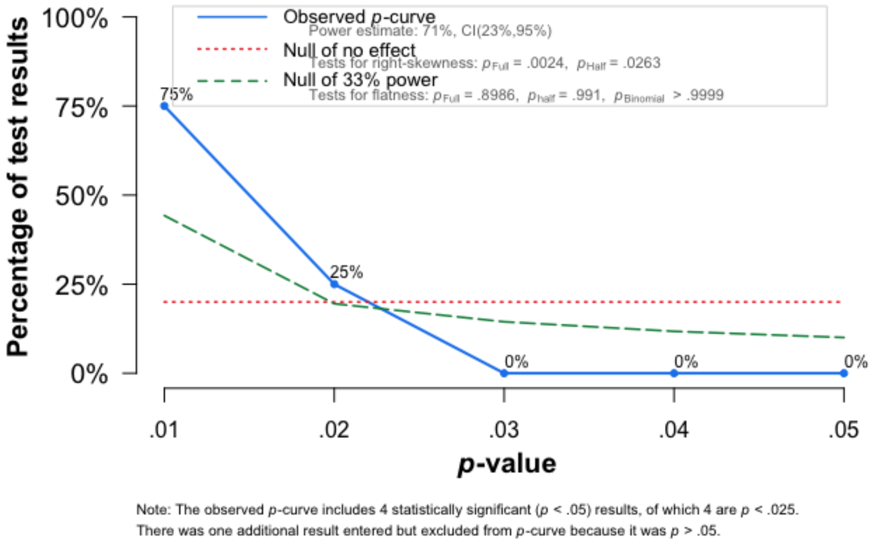

Supplementary Figure 6. P-curve female sexual satisfaction

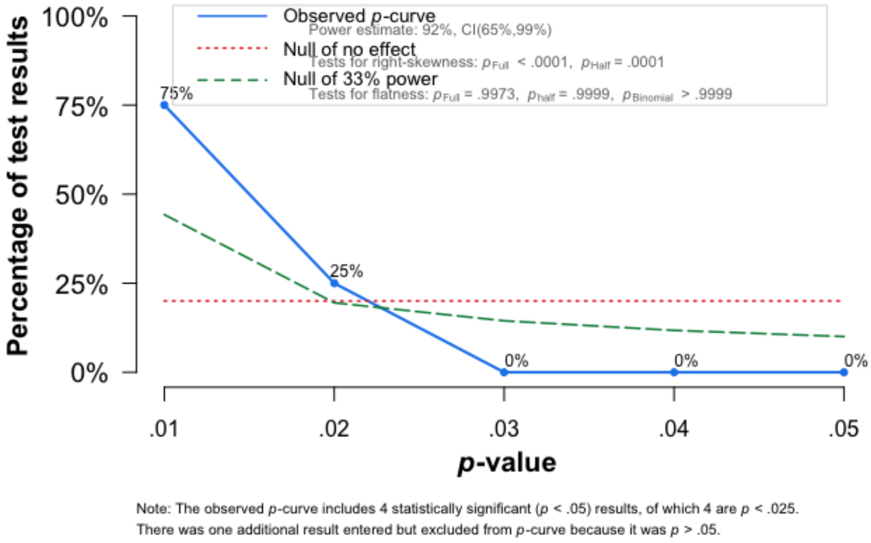

Supplementary Figure 7. Risk of bias plot per study

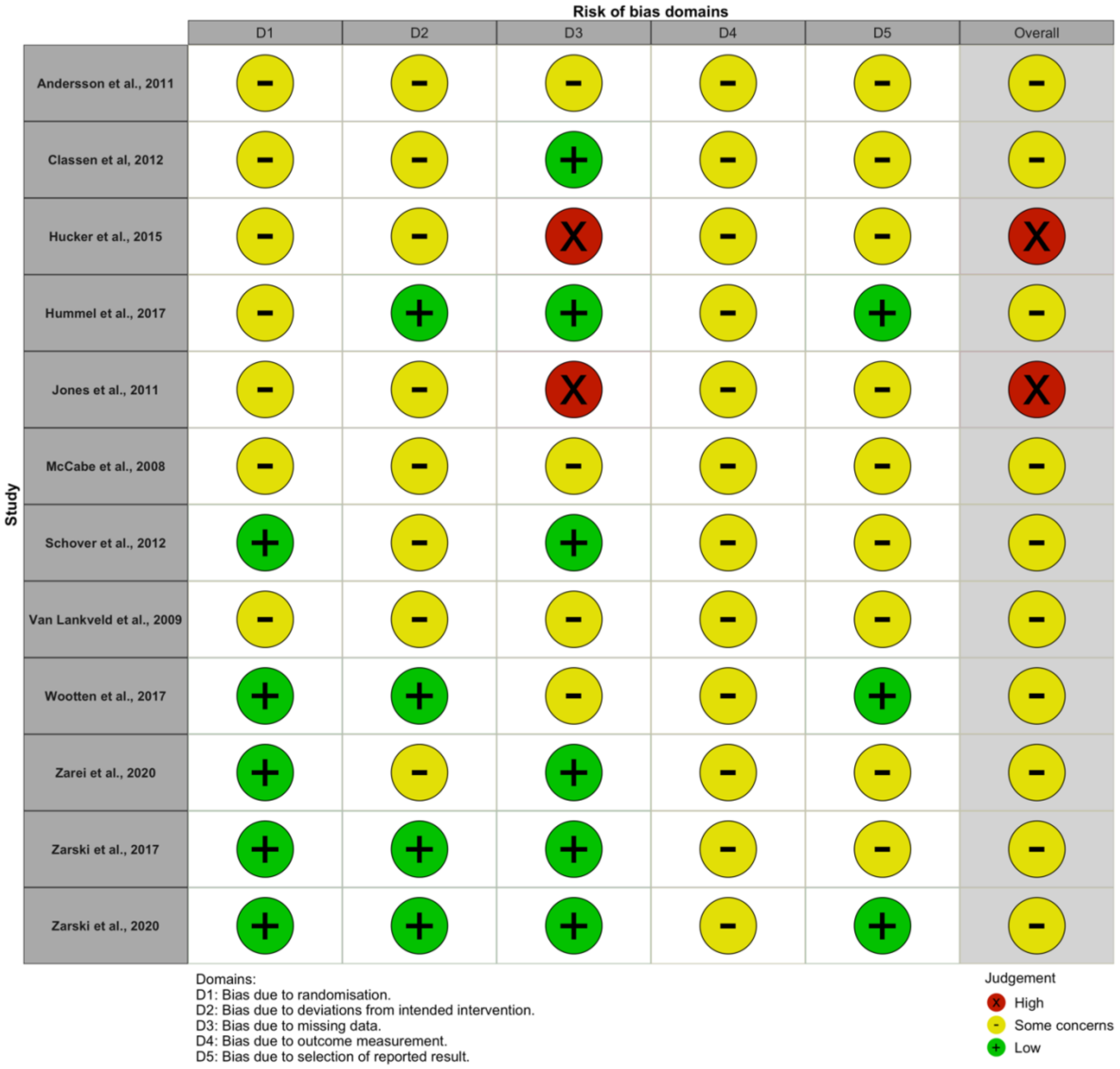

Supplementary Methods: Search String

Cochrane Central Register of Controlled Trials

((“sexual function*”) OR (“sexual difficult*”) OR (“sexual disorder*”) OR (“sexual problem*”) OR (“sexual distress”) OR (“sexual health”) OR (“sexual satisfaction”) OR (“sexual disturb*”) OR (“sexual dysfunct*”) OR (“sexual enjoyment”) OR (“sexual impairment”) OR (psychosexual*) OR (“female sexual function*”) OR (“male sexual function*”) OR (“female sexual difficult*”) OR (“male sexual difficult*”) OR (“female sexual disorder*”) OR (“male sexual disorder*”) OR (“female sexual dysfunction*”) OR (“male sexual dysfunction*”) OR (“erectile dysfunction*”) OR (ejaculation) OR (impotence) OR (vaginismus) OR (”penetration difficult*”) OR (vestibulodynia) OR (vulvodynia) OR (vestibulitis) OR (dyspareunia) OR (“genito-pelvic pain”) OR (“genital pain”) OR (“sexual pain”) OR (“sexual desire”) OR (“sexual interest”) OR (“sexual arousal”) OR (orgasm*) OR (“sexual aversion”)) AND ((internet*) OR (web*) OR (computer*) OR (online*) OR (digital*) OR (tele*) OR (virtual*) OR (electronic) OR (ehealth) OR (e-health) OR (etherap*) OR (e-therap*) OR (icbt) OR (email*) OR (e-mail*) OR (cyber*) OR (mobile*) OR (app*) OR (“mobile application*”) OR (application*) OR (smartphone*)) AND ((therap*) OR (counseling) OR (treat*) OR (consultation) OR (intervention*) OR (“cognitive behavioral therapy”) OR (“cognitive behavioural therapy”) OR (“sex therapy”) OR (self-help*) OR (selfhelp*) OR (treatment*) OR (“psychological intervention”) OR (psychotherap*) OR (program*) OR (care) OR (service) OR (rehabilitation) OR (support)) in Title Abstract Keyword - (Word variations have been searched)

PsycInfo

(“sexual function*”) OR (“sexual difficult*”) OR (“sexual disorder*”) OR (“sexual problem*”) OR (“sexual distress”) OR (“sexual health”) OR (“sexual satisfaction”) OR (“sexual disturb*”) OR (“sexual dysfunct*”) OR (“sexual enjoyment”) OR (“sexual impairment”) OR (psychosexual*) OR (“female sexual function*”) OR (“male sexual function*”) OR (“female sexual difficult*”) OR (“male sexual difficult*”) OR (“female sexual disorder*”) OR (“male sexual disorder*”) OR (“female sexual dysfunction*”) OR (“male sexual dysfunction*”) OR (“erectile dysfunction*”) OR (ejaculation) OR (impotence) OR (vaginismus) OR (”penetration difficult*”) OR (vestibulodynia) OR (vulvodynia) OR (vestibulitis) OR (dyspareunia) OR (“genito-pelvic pain”) OR (“genital pain”) OR (“sexual pain”) OR (“sexual desire”) OR (“sexual interest”) OR (“sexual arousal”) OR (orgasm*) OR (“sexual aversion”) AND (internet*) OR (web*) OR (computer*) OR (online*) OR (digital*) OR (tele*) OR (virtual*) OR (electronic) OR (ehealth) OR (e-health) OR (etherap*) OR (e-therap*) OR (icbt) OR (email*) OR (e-mail*) OR (cyber*) OR (mobile*) OR (app*) OR (“mobile application*”) OR (application*) OR (smartphone*) AND (therap*) OR (counseling) OR (treat*) OR (consultation) OR (intervention*) OR (“cognitive behavioral therapy”) OR (“cognitive behavioural therapy”) OR (“sex therapy”) OR (self-help*) OR (selfhelp*) OR (treatment*) OR (“psychological intervention”) OR (psychotherap*) OR (program*) OR (care) OR (service) OR (rehabilitation) OR (support)

PubMed

((sexual function* [Title/Abstract]) OR (sexual difficult* [Title/Abstract]) OR (sexual disorder* [Title/Abstract]) OR (sexual problem* [Title/Abstract]) OR (sexual distress [Title/Abstract]) OR (sexual health [Title/Abstract]) OR (sexual satisfaction [Title/Abstract]) OR (sexual disturb* [Title/Abstract]) OR (sexual dysfunct* [Title/Abstract]) OR (sexual enjoyment [Title/Abstract]) OR (psychosexual* [Title/Abstract]) OR (female sexual function* [Title/Abstract]) OR (male sexual function* [Title/Abstract]) OR (female sexual difficult* [Title/Abstract]) OR (male sexual difficult* [Title/Abstract]) OR (female sexual disorder* [Title/Abstract]) OR (male sexual disorder* [Title/Abstract]) OR (female sexual dysfunction* [Title/Abstract]) OR (male sexual dysfunction* [Title/Abstract]) OR (erectile dysfunction* [Title/Abstract]) OR (ejaculation [Title/Abstract]) OR

(impotence [Title/Abstract]) OR (vaginismus [Title/Abstract]) OR (penetration difficult* [Title/Abstract]) OR (vestibulodynia [Title/Abstract]) OR (vulvodynia [Title/Abstract]) OR (vestibulitis [Title/Abstract]) OR (dyspareunia [Title/Abstract]) OR (genito-pelvic pain [Title/Abstract]) OR (genital pain [Title/Abstract]) OR (sexual pain [Title/Abstract]) OR (sexual desire [Title/Abstract]) OR (sexual interest [Title/Abstract]) OR (sexual arousal [Title/Abstract]) OR (orgasm* [Title/Abstract]) OR (sexual aversion [Title/Abstract])) AND ((internet* [Title/Abstract]) OR (web* [Title/Abstract]) OR (computer* [Title/Abstract]) OR (online* [Title/Abstract]) OR (digital* [Title/Abstract]) OR (tele* [Title/Abstract]) OR (virtual* [Title/Abstract]) OR (electronic [Title/Abstract]) OR (ehealth [Title/Abstract]) OR (e-health [Title/Abstract]) OR (etherap* [Title/Abstract]) OR (e-therap* [Title/Abstract]) OR (icbt [Title/Abstract]) OR (email* [Title/Abstract]) OR (e-mail* [Title/Abstract]) OR (cyber* [Title/Abstract]) OR (mobile* [Title/Abstract]) OR (app* [Title/Abstract]) OR (mobile application* [Title/Abstract]) OR (application* [Title/Abstract]) OR (smartphone* [Title/Abstract])) AND ((therap* [Title/Abstract]) OR (counseling [Title/Abstract]) OR (treat* [Title/Abstract]) OR (consultation [Title/Abstract]) OR (intervention* [Title/Abstract]) OR(cognitive behavioral therapy [Title/Abstract]) OR (cognitive behavioural therapy [Title/Abstract]) OR (sex therapy [Title/Abstract]) OR (self-help* [Title/Abstract]) OR (selfhelp* [Title/Abstract]) OR (treatment* [Title/Abstract]) OR (psychological intervention [Title/Abstract]) OR (psychotherap* [Title/Abstract]) OR (program* [Title/Abstract]) OR (care [Title/Abstract]) OR (service [Title/Abstract]) OR (rehabilitation [Title/Abstract]) OR (support [Title/Abstract]))

Supplementary References: Overview of included studies

Andersson, E., Walén, C., Hallberg, J., Paxling, B., Dahlin, M., Almlöv, J., … Andersson, G. (2011). A randomized controlled trial of guided internet-delivered cognitive behavioral therapy for erectile dysfunction. *The Journal of Sexual Medicine*, *8*(10), 2800–2809. https://doi.org/10.1111/j.1743-6109.2011.02391.x

Classen, C. C., Chivers, M. L., Urowitz, S., Barbera, L., Wiljer, D., O'Rinn, S., & Ferguson, S. E. (2012). Psychosexual distress in women with gynecologic cancer: A feasibility study of an online support group. *Psycho-Oncology*, *22*(4), 930–935. https://doi.org/10.1002/pon.3058

Hucker, A., & McCabe, M. P. (2015). Incorporating mindfulness and chat groups into an online cognitive behavioral therapy for mixed female sexual problems. *J Sex Res*, *52*(6), 1–13. https://doi.org/10.1080/00224499.2014.888388

Hummel, S. B., Van Lankveld, J. J., Oldenburg, H., Hahn, D., Kieffer, J., Gerritsma, M., … Aaronson, N. (2017). Efficacy of Internet-Based Cognitive Behavioral Therapy in Improving Sexual Functioning of Breast Cancer Survivors: Results of a Randomized Controlled Trial. *Journal of Clinical Oncology*, *35*(12), 1328–1340. https://doi.org/10.1200/jco.2016.69.6021

Jones, L. M., & McCabe, M. P. (2011). The effectiveness of an internet-based psychological treatment program for female sexual dysfunction. *The Journal of Sexual Medicine*, *8*(10), 2781–2792. https://doi.org/10.1111/j.1743-6109.2011.02381.x

McCabe, M. P., Price, E., Piterman, L., & Lording, D. (2008). Evaluation of an internet-based psychological intervention for the treatment of erectile dysfunction. *International Journal of Impotence Research*, *20*(3), 324–330. https://doi.org/10.1038/ijir.2008.3

Schover, L. R., Canada, A. L., Yuan, Y., Sui, D., Neese, L., Jenkins, R., & Rhodes, M. M. (2012). A randomized trial of internet-based versus traditional sexual counseling for couples after localized prostate cancer treatment. *Cancer*, *118*(2), 500–509. https://doi.org/10.1002/cncr.26308

Van Lankveld, J. J., Leusink, P., Van Diest, S., Gijs, L., & Slob, A. K. (2009). Internet-based brief sex therapy for heterosexual men with sexual dysfunctions: A randomized controlled pilot trial. *Journal of Sexual Medicine*, *6*(8), 2224–2236. https://doi.org/10.1111/j.1743-6109.2009.01321.x

Wootten, A. C., Meyer, D., Abbott, J. M., Chisholm, K., Austin, D. W., Klein, B., … Costello, A. J. (2017). An online psychological intervention can improve the sexual satisfaction of men following treatment for localized prostate cancer: Outcomes of a randomised controlled trial evaluating My Road Ahead. *Psycho-Oncology*, *26*(7), 975–981. Retrieved from addie.wootten@mh.org.au

Zarei, F., Rashedi, S., Tavousi, M., Haeri-Mehrizi, A. A., & Maasoumi, R. (2020). A mobile-based educational intervention on sexo-marital life in Iranian men with spinal cord injury: a randomized controlled trial. *Spinal Cord*. https://doi.org/10.1038/s41393-020-0489-4

Zarski, A.-C., Berking, M., & Ebert, D. D. (2021). Efficacy of Internet-Based Treatment for Genito-Pelvic Pain/Penetration Disorder: Results of a Randomized Controlled Trial. *Accepted for Publication in the Journal of Consulting and Clinical Psychology*.

Zarski, A.-C., Berking, M., Fackiner, C., Rosenau, C., & Ebert, D. D. (2017). Internet-based guided self-help for vaginal penetration difficulties: Results of a randomized controlled pilot trial. *The Journal of Sexual Medicine*, *14*(2), 238–254. https://doi.org/10.1016/j.jsxm.2016.12.232
